# Combination of spatial and temporal de-noising and artifact reduction techniques in multi-channel dry EEG

**DOI:** 10.3389/fnins.2025.1576954

**Published:** 2025-06-27

**Authors:** Milana Komosar, Gabriella Tamburro, Uwe Graichen, Silvia Comani, Jens Haueisen

**Affiliations:** ^1^Institute of Biomedical Engineering and Informatics at the Technische Universität Ilmenau, Ilmenau, Germany; ^2^Behavioral Imaging and Neural Dynamics Center, G. d’Annunzio University of Chieti–Pescara, Chieti, Italy; ^3^Department of Neuroscience, Imaging, and Clinical Sciences, G. d’Annunzio University of Chieti–Pescara, Chieti, Italy; ^4^Division Biostatistics and Data Science, Karl Landsteiner University of Health Sciences, Krems an der Donau, Austria

**Keywords:** electroencephalography, magnetoencephalography, spatial harmonic analysis, independent component analysis, biopotential electrode, brain-computer interface

## Abstract

**Introduction:**

Dry electroencephalography (EEG) allows for recording cortical activity in ecological scenarios with a high channel count, but it is often more prone to artifacts as compared to gel-based EEG. Spatial harmonic analysis (SPHARA) and ICA-based methods (Fingerprint and ARCI) have been separately used in previous studies for dry EEG de-noising and physiological artifact reduction. Here, we investigate if the combination of these techniques further improves EEG signal quality. For this purpose, we also introduced an improved version of SPHARA.

**Methods:**

Dry 64-channel EEG was recorded from 11 healthy volunteers during a motor performance paradigm (left and right hand, feet, and tongue movements). EEG signals were denoised separately using Fingerprint + ARCI, SPHARA, a combination of these two methods, and a combination of these two methods including an improved SPHARA version. The improved version of SPHARA includes an additional zeroing of artifactual jumps in single channels before application of SPHARA. The EEG signal quality after application of each denoising method was calculated by means of standard deviation (SD), signal to noise ratio (SNR), and root mean square deviation (RMSD), and a generalized linear mixed effects (GLME) model was used to identify significant changes of these parameters and quantify the changes in the EEG signal quality.

**Results:**

The grand average values of SD improved from 9.76 (reference preprocessed EEG) to 8.28, 7.91, 6.72, and 6.15 μV for Fingerprint + ARCI, SPHARA, Fingerprint + ARCI + SPHARA, and Fingerprint + ARCI + improved SPHARA, respectively. Similarly, the RMSD values improved from 4.65 to 4.82, 6.32, and 6.90 μV, and the SNR values changed from 2.31 to 1.55, 4.08, and 5.56 dB.

**Discussion:**

Our results demonstrate the different performance aspects of Fingerprint + ARCI and SPHARA, artifact reduction and de-noising techniques that complement each other. We also demonstrated that a combination of these techniques yields superior performance in the reduction of artifacts and noise in dry EEG recordings, which can be extended to infant EEG and adult MEG applications.

## 1 Introduction

Electroencephalography (EEG) is a method for monitoring the electrical activity of the brain using electrodes placed on the scalp. It is non-invasive, portable, has high temporal resolution, and has lower costs than other brain imaging techniques (Mannan et al., 2018; Knösche and Haueisen, 2022). EEG is widely used in clinical settings to diagnose and monitor neurological and psychiatric disorders (Livint Popa et al., 2020). Beyond clinical use, EEG is widely applied in research and brain-computer interface (BCI) development (Lee et al., 2019), enabling direct interaction between the brain and external devices.

While EEG has numerous advantages, its effectiveness heavily depends on the quality of the recorded signal, which is often affected by artifacts. Artifacts are defined as events overlying the measured brain activity. They can arise from a variety of sources, including physiological sources such as eye movements, muscle and cardiac activity, as well as non-physiological sources such as environmental or instrumental interference and electrode or cable movements (Jiang et al., 2019; Rashmi and Shantala, 2022; Roy, 2022). Consequently, artifact and noise reduction is an integral part of EEG data analysis (Mannan et al., 2018; Seok et al., 2021).

A large variety of artifact and noise reduction methods and pipelines have been proposed (Albera et al., 2012; Daly et al., 2015; Desjardins et al., 2021; Mathe et al., 2021; Roy, 2022). Traditional methods include temporal filtering, drift compensation, and decomposition methods such as blind source separation (BSS) (Hyvärinen and Oja, 2000; Urigüen and Garcia-Zapirain, 2015). Independent component analysis (ICA) is a popular technique that decomposes EEG signals into statistically independent components, allowing isolation and removal of artifacts (Jung et al., 2000; Delorme and Makeig, 2004). Wavelet Transform is particularly effective in removing artifacts across frequency bands (Krishnaveni et al., 2006; Turnip and Pardede, 2017), while adaptive filtering can use reference signals like electrooculography (EOG) for artifact removal (Sweeney et al., 2012; Hua, 2020–2020). Convolutional neural networks (CNNs) have also been proposed for artifact reduction (Nejedly et al., 2019; Bao et al., 2022). Besides many efforts in developing EEG cleaning procedures, there is no single specific solution for removing all types of artifacts (Jiang et al., 2019; Rashmi and Shantala, 2022).

Beyond the standard gel-based EEG systems, dry EEG introduces advantages such as self-applicability and rapid setups, which prospectively make them preferable for several experimental and clinical applications (Fiedler et al., 2015). However, dry EEG is more susceptible to artifacts, especially those caused by movements (di Fronso et al., 2019; Komosar et al., 2022). While many artifact reduction methods have been developed and tested for conventional gel-based EEG, the different mechanical properties of dry EEG electrodes introduce additional challenges. In conventional systems, the gel reduces the electrode-skin impedance and serves as a mechanical buffer, providing higher mechanical stabilization during electrode movements. In contrast, dry EEG lacks this gel-based stabilization. As a result, gel-based EEG typically has less pronounced artifacts compared to dry EEG (Knösche and Haueisen, 2022; Fiedler et al., 2023). The reduction of movement artifacts in EEG is especially important for naturalistic studies but still challenging. Due to the above-mentioned mechanics of gel-based and dry EEG systems, these artifacts pose even more challenges for their reduction in dry EEG systems (Kilicarslan and Contreras Vidal, 2019). At the same time, there is great potential for the use of dry EEG in human movement science, particularly due to its simplified applicability and shorter preparation time, which facilitate efficient data collection in dynamic and real-world settings (Comani et al., 2021). Despite the growing need for dry EEG systems, dedicated artifact reduction pipelines are still scarce.

Here, we analyze dry EEG signals recorded during the performance of specific body movements. For such data, no artifact reduction pipelines have been explicitly proposed so far. In line with the recent suggestion to use combinations of artifact reduction methods (Gorjan et al., 2022), we propose a pipeline using a combination of cleaning methods for dry EEG based on temporal, spectral, statistical, and spatial properties. Specifically, we combine the ICA-based methods Fingerprint and ARCI (Tamburro et al., 2018; Tamburro et al., 2019) and the SPatial HARmonic Analysis (SPHARA) (Graichen et al., 2015), both of which have been used separately for dry EEG artifact reduction and de-noising. The ICA-based methods have been specifically developed to remove physiological artifacts (eye blinks, eye movements, muscle artifacts, pulse and cardiac interferences), whereas spatial filtering has been used for SNR improvement and dimensionality reduction (Graichen et al., 2015; Das et al., 2019; Banville et al., 2021; Cohen, 2021). Consequently, we aim to assess the artifact and noise reduction performance of the combination of these methods (i.e., Fingerprint + ARCI and SPHARA) in dry EEG recordings. To this end, we also introduce an improved version of SPHARA.

## 2 Materials

### 2.1 Study group

Eleven healthy volunteers with an average age of 25 years participated in the study. All participants gave written informed consent before the experiment. None of them had any neurological disorder, and none of them had prior experience with experiments of this type (BCI-naïve volunteers). The study procedure is in line with the Declaration of Helsinki and was approved by the ethics committee of the Faculty of Medicine of the Friedrich-Schiller-University Jena, Germany.

### 2.2 Equipment

**FIGURE 1:**
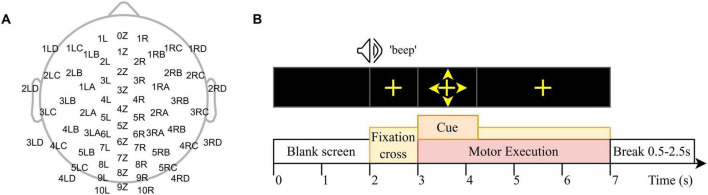
**(A)** The topographical representation of the 64-channels layout of the dry EEG cap and **(B)** the experimental paradigm.

The EEG was recorded using an eego™ amplifier and a 64-channel EEG cap with dry PU/Ag/AgCl electrodes (waveguard™touch, emagine Medical Imaging Solutions GmbH, Berlin, Germany). The channel layout is shown in Figure 1A. Gel-based ground and reference electrodes were placed on the left and right mastoids, and their impedances were kept below 50 kΩ. All channels were sampled at a rate of 1,024 Hz.

### 2.3 Paradigm

A motor execution paradigm adopted from Pfurtscheller et al. (2006) was implemented using the eevoke software (eemagine Medical Imaging Solutions GmbH, Berlin, Germany), as shown in Figure 1B. The volunteers were instructed to sit comfortably on a chair in front of a screen. For the first 2 s, the screen was black. After this, an acoustic signal (“beep”) appeared as well as a cross in the middle of the screen, and the volunteers had to fixate the cross for the next 5 s. After 1 s, an arrow appeared next to the cross, pointing either left, right, up, or down indicating the left hand (L), right hand (R), tongue (T), or both feet (F) movement task. The arrow was presented on the screen for 1.25 s, after which only the cross was presented. The volunteers were asked to start executing the movement when they saw an arrow on the screen and to continue performing the motor task until the cross disappeared. The movements were explained to be performed in a calm manner. For hand movements, the volunteers were instructed to touch each finger of the respective hand with the thumb, repetitively as long as the cross was presented. Tongue movements involved moving the tongue up and down or in circular motions within the oral cavity. Similarly, foot movements were performed by moving the feet up and down. Each trial lasted 7 s, with an inter-trial interval ranging from 0.5 to 2.5 s. The experiment consisted of six runs, with short breaks in between to allow the volunteer to relax. Each run contained 10 repetitions of each task. In total, 60 trials of each task were recorded.

## 3 Methods

### 3.1 EEG data analysis

**FIGURE 2:**
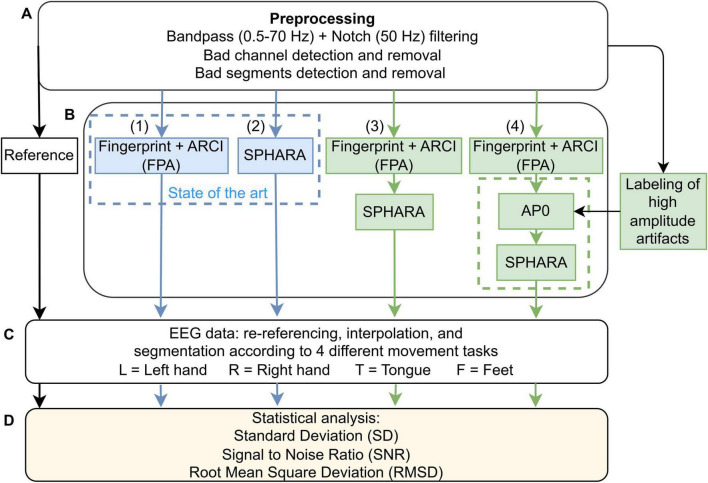
Flowchart of the data processing pipeline for EEG data cleaning, including phases A—preprocessing, B—application of different methods, C—data re-referencing, interpolation, and segmentation according to the movement tasks, and D—statistical analysis. The standard deviation (SD) is calculated for preprocessed signal, (1) FPA, (2) SPHARA, (3) FPA + SPHARA, and (4) FPA + AP0 + SPHARA. The signal to noise ratio (SNR) and root mean square deviation (RMSD) are calculated by comparing each of the four methods (1, 2, 3, and 4) with the preprocessed signal (Ref).

The signal analysis was divided into four phases, as shown in Figure 2. In the first phase (A), EEG data were preprocessed to provide a base for further analysis. In the second phase (B), four EEG cleaning methods were applied to the preprocessed dry EEG signals: (1) Fingerprint + ARCI; (2) SPHARA; (3) a combination of Fingerprint + ARCI + SPHARA; (4) Fingerprint + ARCI + improved SPHARA. The last two methods were tested for the first time. Improved SPHARA includes an additional step, which is called AP0. In AP0, artifactual periods, i.e., periods of high amplitude signal jumps—which occur occasionally in the signal, were set to 0 V. AP0 allowed an improved performance of SPHARA, as explained below. In the third phase (C), the clean EEG data were prepared for the next phase of evaluating the effectiveness of the methods in artifact and noise reduction. In the fourth phase (D), three evaluation parameters were calculated for all methods and their differences were statistically tested: standard deviation (SD), root mean square deviation (RMSD), and signal to noise ratio (SNR). The analysis was performed in MATLAB (release MATLAB R2021a; MathWorks, Natick, MA, USA) and Python 3.9. The description of the signal preprocessing, the individual and combined methods, and the signal quality evaluation is given below.

#### 3.1.1 Phase A: preprocessing

During phase A the EEG data were preprocessed using a finite impulse response (FIR) bandpass filter between 0.5 Hz and 70 Hz implemented in EEGLAB (release v2021.1) (Delorme and Makeig, 2004). A notch filter at 50 Hz was applied to reduce power line noise. Then, the filtered EEG data were visually inspected, and signals recorded from non-operating EEG electrodes, as well as trials with disturbances in more than 40% of the channels, were excluded from further analysis. Channels with isoelectric lines or high amplitudes for more than 70% of the time were considered as bad channels and removed from further analysis.

The preprocessed signal provided the basis for the application of the other methods and served as a reference signal (Reference; Ref) for evaluating their performance.

#### 3.1.2 Phase B: cleaning methods

##### 3.1.2.1 FPA: Fingerprint + Automatic Removal of Cardiac Interference (ARCI)

These two methods were designed to automatically remove physiological artifacts (eye blink, eye movement, cardiac and pulse interference, myogenic activity) based on the evaluation of features of the independent components (ICs) into which the EEG signals are decomposed. The EEG datasets were pre-whitened by principal components analysis (PCA; Delorme et al., 2007) and decomposed into 50 ICs using the extended Infomax algorithm, which has been proven to better separate signal sources that may exhibit super-Gaussian and sub-Gaussian distributions (Bell and Sejnowski, 1995; Lee et al., 1999). All datasets were decomposed into 50 ICs to ensure comparability across the preprocessed EEG recordings, as the number of retained channels varied after preprocessing due to the removal of bad channels. Given that the number of retained EEG channels was always higher than 50, and based on prior evidence that artifactual ICs can be successfully identified with the Fingerprint method regardless of the decomposition level (Tamburro et al., 2018), we decided to decompose each EEG dataset in 50 ICs.

The automated classification of artifactual ICs was performed by applying the Fingerprint method to detect and remove ICs containing eye blinks, eye movements, and myogenic artifacts (Stone et al., 2018), whereas the ARCI approach (Tamburro et al., 2019) was used to classify the ICs containing cardiac-related artifacts, including pulse interference. The Fingerprint method employs a genetic algorithm to identify, for each physiological artifact (i.e., eye blinks, eye movements, myogenic artifacts), the optimal feature set for an automated support vector machine (SVM) classifier (Stone et al., 2018). Three classifiers are sequentially applied to the ICs, automatically classifying the ICs containing eye blinks, eye movements, and muscle interference. The ARCI approach evaluates time and frequency features of the separated ICs to automatically identify the ICs containing cardiac and pulse interference (Tamburro et al., 2019).

For each EEG dataset: (1) the ICs classified as artifactual by the Fingerprint method and the ARCI approach, hence those related to the five physiological artifacts (eye blinks, eye movement, myogenic activity, electrical and pulsatile activity of the heart) were disregarded and (2) the retained ICs were reprojected onto the sensor space to reconstruct artifact-reduced EEG signals. Fingerprint and ARCI EEG data processing and decomposition were performed using the EEGLAB toolbox (v. 13.6.5b; Delorme and Makeig, 2004).

##### 3.1.2.2 SPHARA

SPHARA, short for SPherical HARmonic Analysis, is a method that extends classical spatial Fourier analysis to EEG sensors that are non-uniformly positioned over the surface of the head. It offers a tool for spatial filtering while preserving the phase properties of EEG time series. A detailed description of the method is available in Graichen et al. (2015, 2019). Here, we provide a brief overview of its principles and application.

At the core of SPHARA are basis functions (BFs), which are mathematical representations used to describe the spatial properties of EEG signals. The BFs are determined by the eigenanalysis of the discrete Laplace-Beltrami operator, which is defined on a triangular mesh specified by the spatial sampling points (the positions of the sensors on the head surface). A key advantage of SPHARA is that BFs can be computed prior to EEG data acquisition, which distinguishes it from other linear decomposition methods, such as PCA or ICA. It was demonstrated that SPHARA-based spatial filtering does not affect the phase properties of the EEG time series (Graichen et al., 2019).

In this paper, we used SPHARA with a finite element method (FEM) discretization (Graichen et al., 2015). For the reconstruction of the EEG data, a subset of BFs was selected to retain 95% of the total signal power. The spatial filtering was performed using a Butterworth low-pass filter of order 2. EEG data processing was performed using SpharaPy, a Python implementation of SPHARA (Graichen et al., 2019).

##### 3.1.2.3 FPA + SPHARA: Fingerprint + ARCI and SPHARA

As a third cleaning method, we used a sequential combination of Fingerprint + ARCI and SPHARA to clean the EEG signals. The parameters used in this method were identical to those described above for methods (1) and (2) to ensure consistency when comparing cleaning performance between methods. The primary objective of this approach was to evaluate how the integration of cleaning techniques could improve EEG signal quality. By combining these methods, we aimed to access their joint impact on artifact and noise reduction, while preserving the EEG signal, thereby offering a more comprehensive approach to EEG data cleaning.

##### 3.1.2.4 FPA + AP0 + SPHARA: Fingerprint + ARCI and improved SPHARA

In this method, we successively applied Fingerprint + ARCI and an improved version of SPHARA to clean the EEG signals. The improved version of SPHARA involved an additional process called artifactual period zeroing (AP0), in which high amplitude signal jumps were set to 0 V before applying SPHARA. This approach was necessary because, during the EEG review, we observed transient, high amplitude jumps in the time series data affecting single channels or groups of a few nearby channels. These jumps could easily exceed hundreds of μV and might be related to movement artifacts (Shahbakhti et al., 2021). Such high amplitude jumps can negatively affect any spatial filter, including SPHARA, as they cause the artifact to spread spatially, but with reduced amplitude. To prevent such spreading, AP0 was introduced. The AP0 introduces the zeroing of such high amplitude jumps. This was done based on visual inspection and manual labeling. The AP0 periods are those which largely exceeded the normal range of EEG (transient high amplitude signal deflections, typically exceeding 150 μV). The AP0 period was labeled when the EEG signal started to deviate from the normal amplitude range with the onset marked at least 200 ms before the occurrence of the high amplitude jump. The labeled AP0 period ended after the EEG signal stabilized within the range of ± 80 μV for at least 200 ms. These identified AP0 periods were set to 0 V. A 0.5 s Hann window was used to ensure smooth transitions between regular EEG signal and the zeroed period. Following the AP0 processing step, SPHARA was applied and the EEG signal in the affected channel was interpolated from the surrounding channels during these AP0 periods.

The performance of the Fingerprint and ARCI methods was not affected by these high amplitude jumps in the EEG signal. Therefore, the AP0 + SPHARA procedure was performed after having applied the Fingerprint + ARCI methods.

#### 3.1.3 Phase C: clean EEG data preparation

In this phase, the clean EEG data were re-referenced to common average, and the EEG channels removed during preprocessing were interpolated using spline interpolation. Within this phase, the EEG data were also segmented into four data groups according to the tasks performed: L, R, T and F, corresponding to left hand, right hand, tongue and foot movements, respectively.

#### 3.1.4 Phase D: methods’ performance evaluation

##### 3.1.4.1 Evaluation metrics

Besides visual inspection of the data (Mannan et al., 2018), we used standard deviation (SD) (Dovedi et al., 2022), signal to noise ratio (SNR) (Väisänen and Malmivuo, 2009; Urigüen and Garcia-Zapirain, 2015; Bao et al., 2022), and root mean square deviation (RMSD) (Arpaia et al., 2022) as quantitative metrics to assess differences in EEG signals before and after the application of the cleaning methods. To evaluate the quality of the EEG signals reconstructed after the cleaning methods, the level of contamination in the signal before and after the cleaning was compared by calculating the SNR across the entire length of the recorded signal (M samples). For this analysis, we adopted a definition of SNR similar to that used by Tamburro et al. (2018), where the signal was the specific physiological artifact to be removed (typically with a larger amplitude than clean EEG signals) and the noise was the clean EEG signal after artifact removal. With this approach, a reduction in SNR in the noisy EEG segment indicated a good suppression of the artifact. In our case, the preprocessed signal prior to artifact reduction (*EEG*_*Ref*_) served as the reference, while the noise is defined as the signal after applying one of the specific cleaning method (EEG*_*mx*_*). Under this formulation, an increase in SNR is an indicator of effective artifact and noise reduction.


(1) $$\mathrm{SNR}=10\,l\,o\,g_{10}\,\left(\frac{\sum_{i=1}^{M}{\mathrm{EEG}}_{\mathrm{Ref}}\,{\left(i\right)}^{2}}{\sum_{i=1}^{M}{\mathrm{EEG}}_{m\,x}\,{\left(i\right)}^{2}}\right)$$

Please note that our definition of SNR in Equation (1) differs from the conventional definition of the signal to noise ratio (SNR), which typically requires a clear separation between signal and noise components. In our case, as the ground truth “clean” signal is not available, we instead use the power ratio between the reference EEG (prior to cleaning) and the processed EEG as a relative measure, with the intent to quantify the attenuation introduced by the artifact and noise reduction methods.

The metric in Equation (1) can be interpreted as the logarithmic ratio of total power (in the RMS sense) before and after processing. This represents the overall change in signal magnitude. Instead of using this metric as a stand-alone indicator of cleaning quality, we present it alongside SD and RMSD to better contextualize the degree of signal change across different methods. The SNR metric is intended more for relative comparison than for providing an absolute measure of signal preservation.

We used RMSD as a measure of the difference between the reference signal (EEG_*Ref*_) and the signal cleaned by each of the methods presented here (EEG*_*mx*_*). It was calculated over the entire length of the recorded signal (M samples). An increase in RMSD indicates larger differences between two compared signals, which, in our case, suggests that artifacts and noise have been effectively removed from the EEG signal.


(2) $$\mathrm{RMSD}=\sqrt{\frac{1}{M}\,\sum_{i=1}^{M}{\left({\mathrm{EEG}}_{m\,x}\,\left(i\right)-{\mathrm{EEG}}_{\mathrm{Ref}}\,\left(i\right)\right)}^{2}}$$

##### 3.1.4.2 Statistical analysis

A generalized linear mixed effects (GLME) model (Koerner and Zhang, 2017; Frömer et al., 2018) was used to test for statistical differences in the effects of different cleaning methods and different motor execution tasks on the standard deviation (SD), signal to noise ratio (SNR), and root mean square deviation (RMSD) of the EEG signals. The model consisted of fixed effects: Ref, FPA, SPHARA, FPA + SPHARA, FPA + AP0 + SPHARA and four tasks (L, R, T, F), while random effects accounted for individual channels and volunteers. This approach accounts for dependencies in the data. The model, using a log-link function and appropriate distributions, adjusts for non-normally distributed EEG parameters, enabling quantification of method and task effects on signal quality. The results of the GLME test give the estimated values of the observed parameters for the methods and tasks compared to the reference method (preprocessed signal = Ref) and the left hand (L) movement task. For the direct comparison of the effects of channels and volunteers and model error, the results were back-transformed. A *post hoc* F-test, adjusted by Bonferroni correction, explored remaining differences between all methods and tasks that were not explicitly covered in the initial GLME analysis, with corrected *p*-values of 0.0056 for SD, and 0.0083 for SNR and RMSD. All statistical tests were performed in MATLAB (release MATLAB R2024a; MathWorks, Natick, MA, USA).

## 4 Results

### 4.1 Total duration of labeled AP0s across datasets

The average total analyzed EEG duration for each volunteer was 30 h, calculated as the sum across all channels. On average, each volunteer dataset contained approximately 24 min of EEG data labeled as artifactual jump periods. This corresponds to 0.4 h of data labeled as high amplitude jump periods. Therefore, the relative duration of artifactual high amplitude jump periods was 1.3 ± 0.9% of the total EEG signal duration. Supplementary Table 1 summarizes the total duration of all analyzed EEG data and the total duration of all artifactual jump periods set to zero Volts for each volunteer, aggregated across all channels and experimental tasks.

### 4.2 Qualitative method comparisons

**FIGURE 3:**
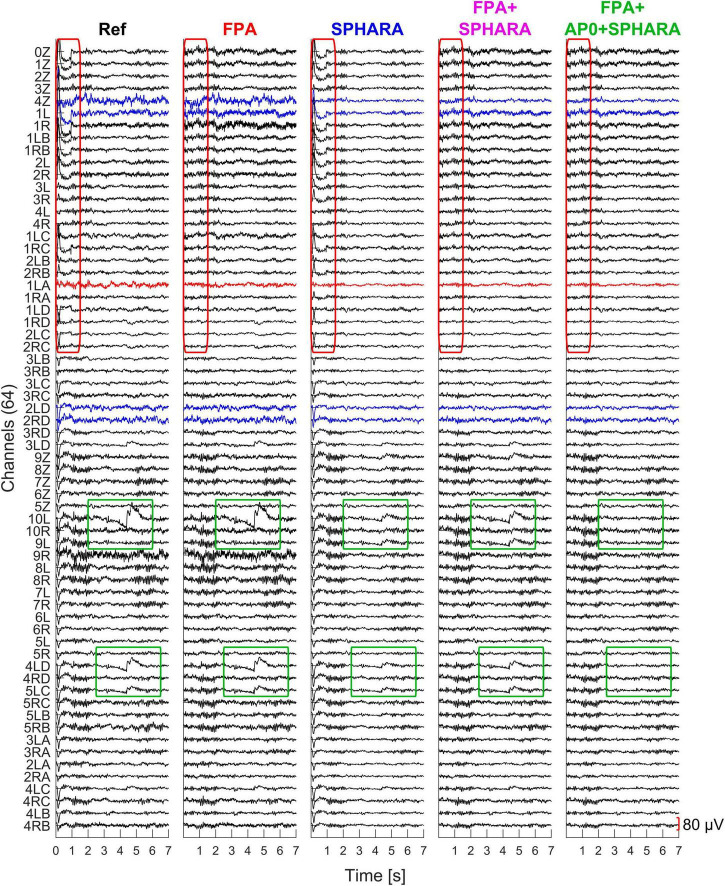
Example of 7-s EEG epoch processed with the cleaning methods. Columns from left to right represent the preprocessed signal (Ref), followed by the signal after applying FPA, SPHARA, FPA + SPHARA, and FPA + AP0 + SPHARA methods. The red rectangle highlights eye movement artifacts present in the preprocessed (Ref) and in the SPHARA cleaned signal, which were removed by the FPA method. A pulse artifact in channel 1LA, also marked in red, was eliminated with FPA. Noise in channels 4Z, 1L, 2LD, and 2RD was reduced with SPHARA. Jump artifacts, marked by green rectangles in channels 10L, 4LD, and 5LC, were attenuated by SPHARA but caused smearing into adjacent channels. These artifacts were removed when AP0 was applied prior to SPHARA (FPA + AP0 + SPHARA column).

Figure 3 shows an example of a 7-s EEG epoch preprocessed within the frequency range of 0.5–70 Hz, followed by artifact reduction and de-noising using the different cleaning methods. Each column represents the signals of the 64 dry EEG channels after applying one of methods: from left to right, the columns show the preprocessed signal (Ref, which serves as the reference) and the signal cleaned by FPA, by SPHARA, by FPA + SPHARA, and by FPA + AP0 + SPHARA. This figure allows for a direct visual comparison of the effects of each method on the same EEG signal segment, highlighting their relative performance in artifact and noise reduction. Eye movement artifacts are marked in red boxes in Figure 3 and are prominent in channels 0Z–2RC in the preprocessed signal (Ref) and after the application of SPHARA. In all other cases, when the Fingerprint + ARCI (FPA) method was applied, these artifacts were effectively reduced. Pulse artifacts are observed in channel 1LA and marked in red. These artifacts were efficiently removed by the FPA method, which successfully targets and removes physiological signals like cardiac interference. SPHARA, on the other hand, excels at removing uncorrelated noise present in channels such as 4Z, 1L, 2LD, and 2RD, which are marked in blue, significantly improving signal clarity in these regions. Jump artifacts, which are high amplitude transients caused, for example, by electrode movements, are identified in channels 10L, 4LD, and 5LC (included in green boxes in the Figure 3). SPHARA attenuates these artifacts by reducing their amplitude, but the residual energy of the artifacts causes them to smear into adjacent channels, as seen in channel 9L. By introducing the AP0 procedure, where such high amplitude artifacts are set to zero Volts before SPHARA is applied, these artifacts are not only attenuated but completely removed, as shown in the FPA + AP0 + SPHARA column. This detailed example illustrates the incremental improvements achieved by combining Fingerprint + ARCI and SPHARA artifact and noise reduction methods. The progressive removal of artifacts and noise, culminating in the cleanest signal with FPA + AP0 + SPHARA, highlights the importance of integrating Fingerprint + ARCI and SPHARA cleaning techniques for optimal EEG data quality.

### 4.3 Parametric evaluation of signal changes

#### 4.3.1 Standard deviation (SD) of EEG signals

The grand average standard deviation values of the EEG data are 9.76, 8.28, 7.91, 6.72, and 6.15 μV, for the preprocessed signal, FPA, SPHARA, FPA + SPHARA, and FPA + AP0 + SPHARA methods, respectively. The statistical analysis of the EEG signals’ standard deviation showed a trend of significant reduction in SD with every more advanced cleaning method applied. Task comparisons reveal distinct patterns in SD variability: right hand movement (R) exhibits a significantly lower SD, while tongue movement (T) shows a higher SD compared to left-hand movement (L). Foot movement (F) does not show a significant difference from L, with a *p*-value of 0.186. The results indicate that the standard deviation of the SD across both random effects, volunteers and channels, is 1.157 μV after back-transformation of the data. The residual error of the GLME model is 1.411 μV after back-transformation of the data and shows the variability in the SD that remains unexplained after considering both fixed and random effects. Additionally, an F-test confirmed significant differences in SD across the methods (FPA, SPHARA, FPA + SPHARA, FPA + AP0 + SPHARA) and tasks (R, T, F), with *p*-values below 0.0056. Detailed results of the GLME analysis, including statistical parameters, are included in Supplementary Table 2.

#### 4.3.2 Signal to noise ratio (SNR)

**FIGURE 4:**
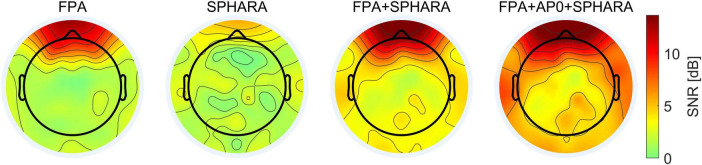
Grand average topographic maps of the channel SNR for the four cleaning methods (FPA, SPHARA, FPA + SPHARA, FPA + AP0 + SPHARA).

The average SNR values (see Equation 1 and explanation in section “3.1.4.1 Evaluation metrics”) calculated for the FPA, SPHARA, FPA + SPHARA, and FPA + AP0 + SPHARA methods are 2.31, 1.55, 4.08, and 5.56 dB, respectively. Figure 4 depicts the topographic maps of the channel SNR for the four artifact and noise reduction methods. The SNR was calculated by comparing the signal obtained after each cleaning method (FPA, SPHARA, FPA + SPHARA, FPA + AP0 + SPHARA) to the preprocessed signal (Ref). Each map represents SNR values distributed over the scalp surface and averaged across all volunteers. The high SNR in the frontal regions for the FPA, FPA + SPHARA, and FPA + AP0 + SPHARA methods indicates significant changes in the EEG signal in these frontal electrodes. This result can be attributed to the specific characteristics of the FPA method for artifact reduction and the nature of the processed EEG data. Specifically, the analyzed EEG recordings contain numerous eye movement and eye blink artifacts. Given that the FPA method is specialized in removing these interferences, its application leads to a remarkable SNR increase. The SPHARA method performs a general de-noising of the EEG and removes disturbances that may appear across various electrodes. For our EEG data, where electrodes are not systematically affected by high amplitude artifacts, and the topography represents an average across all volunteers, the result is a fairly uniform distribution of SNR values across the head surface. In the SNR result for FPA + AP0 + SPHARA, a further increase in SNR is observed in the temporal (area around the ears) and occipital areas.

The statistical analysis shows that the average SNR for SPHARA and the combination of methods (FPA + SPHARA and FPA + AP0 + SPHARA) is, respectively, lower and higher than for FPA. This trend aligns with the SNR topographic maps shown in Figure 4, where the largest SNR improvement is observed for the combination of FPA + AP0 + SPHARA, particularly in the frontal and prefrontal regions. These areas are primarily affected by eye blink and eye movement artifacts, which are effectively reduced by the FPA method. Task-wise, an increase in average SNR is noted for R and F trials compared to L, while no significant difference is observed between T and L trials (*p* = 0.865). The results also show that the standard deviation of the SNR is 0.777 dB between volunteers and 1.531 dB between channels. The residual error of the GLME model is 2.954 dB and shows the SNR variability that remains unexplained after considering both fixed and random effects. The F-test showed significant differences in SNR between all remaining methods (SPHARA, FPA + SPHARA, FPA + AP0 + SPHARA) and experimental tasks (R, T, F), except when comparing the SNR for the R and F tasks, where the *p*-value is 0.385. Detailed results of the GLME analysis, including statistical parameters, are available in Supplementary Table 3.

#### 4.3.3 Root mean square deviation (RMSD)

The average RMSD values calculated for the FPA, SPHARA, FPA + SPHARA, and FPA + AP0 + SPHARA methods are 4.65, 4.82, 6.32, and 6.90 μV, respectively. Therefore, the RMSD increases progressively with every more advanced cleaning method applied, with the highest average RMSD after application of the FPA + AP0 + SPHARA method. These increases in average RMSD are statistically significant for all methods compared to FPA, as indicated by *p*-values below 0.05.

**FIGURE 5:**
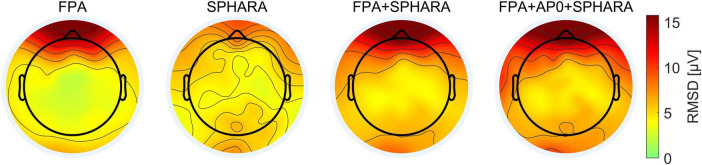
Grand average topographic maps of channel RMSD for the four cleaning methods (FPA, SPHARA, FPA + SPHARA, FPA + AP0 + SPHARA).

Figure 5 shows the topographic maps of the channel RMSD for the four artifact and noise reduction methods (FPA, SPHARA, FPA + SPHARA, FPA + AP0 + SPHARA). Although the average RMSD for SPHARA is higher than for FPA, the distribution of RMSD values across channels confirms the higher effectiveness of FPA in removing artifacts related to ocular movements and the higher performance of SPHARA in the general reduction of noise in all channels. Task-wise, higher average RMSD values are observed for T and F tasks compared to L tasks, whereas the difference between R and L tasks is not significant (*p*-value = 0.592). The results indicate that the standard deviation of the RMSD across the random effects, volunteers and channels, is 1.317 μV and 1.198 μV after back-transformation of the data. The residual error of the GLME model is 1.611 μV after back-transformation of the data and shows the variability in the RMSD that remains unexplained after considering both fixed and random effects. The F-test confirmed significant differences in RMSD between all remaining methods (SPHARA, FPA + SPHARA, FPA + AP0 + SPHARA) and experimental tasks (R, T, F), with *p*-values below 0.0083. Detailed statistical results of the GLME analysis can be found in Supplementary Table 4.

## 5 Discussion

**FIGURE 6:**
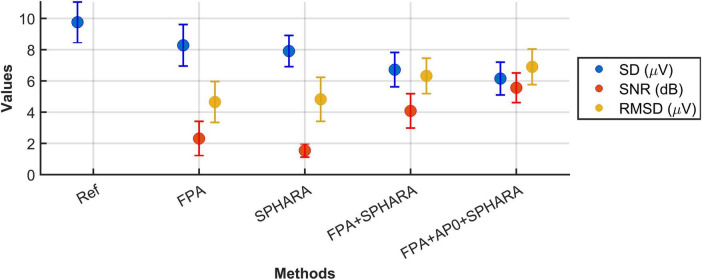
Comparative analysis of EEG signals’ standard deviation (SD), signal to noise ratio (SNR), and root mean square deviation (RMSD) across all tested methods: preprocessed signal (Ref), Fingerprint + ARCI (FPA), SPHARA, FPA + SPHARA, FPA + AP0 + SPHARA. The scatter points represent the average values, while the error bars indicate the standard deviation across the datasets.

We applied Fingerprint + ARCI and SPHARA artifact and noise reduction methods as well as their combination to clean dry EEG data acquired during the performance of four different motor tasks. We added a new processing step to the SPHARA method, setting the high amplitude artifacts in single channels to zero Volts. The combination of methods yielded the best cleaning performance, as indicated by the lower standard deviation, the higher SNR, and the higher RMSD. Figure 6 illustrates the changes in the average values of all metrics for the reference signal, the single and the combined methods. The higher average SNR obtained when applying FPA than when applying SPHARA can be explained by the fact that the recorded EEG data had a considerable number of physiological artifacts, and that FPA was developed to specifically detect and remove this type of artifacts, therefore making a higher contribution in cleaning the EEG signals and increasing the SNR, i.e., reducing signal amplitude. Although the SD and SNR values for FPA + SPHARA and FPA + AP0 + SPHARA appear visually similar in Figure 6, *post hoc* F-tests showed statistically significant differences for both metrics: SD [*F*(1, 14,072) = 83.96, *p* = 5.75 * 10^20^] and SNR [*F*(1, 11,257) = 51.98, *p* = 5.98 * 10^13^]. These findings indicate that the inclusion of the AP0 step before SPHARA resulted in a measurable improvement in signal quality compared to the FPA + SPHARA combination alone.

Task-wise, the largest differences observed across all parameters regarded tongue movements (T). EEG is particularly susceptible to tongue movement artifacts because they can lead to incidental contractions of the facial muscles, causing movements of the EEG cap and/or electrodes. In fact, these trials were more affected by artifacts than the trials involving left hand (L), right hand (R), and feet (F) movements. The visual representations in Supplementary Figure 1 align with these observations, further highlighting the high standard deviation in tongue movement (T) trials. The statistical analysis confirmed significantly higher standard deviations in the data related to tongue movements (Supplementary Table 2). Consequently, the tongue movement data also had the highest RMSD values (Supplementary Table 4). These findings suggest that cleaning had the most pronounced impact on tongue movement (T) trials.

The GLME models for RMSD reveal that the random effect of the channel has a higher standard deviation when compared to the random effect of the volunteer. This suggests that the effectiveness of the methods used for removing artifacts can vary across channels because it is influenced by the nature of the artifacts affecting specific channels. For instance, frontal channels are more susceptible to physiological artifacts such as eye blinks and eye movements and in this case FPA resulted to be more effective than SPHARA in reducing artifacts in those channels, whereas other channels are more prone to be randomly affected by large amplitude artifacts and in this case SPHARA outperformed FPA.

To reflect established conventions in EEG preprocessing research, we report SD, SNR, and RMSD as general descriptors of signal quality after de-noising. These values provide a contextual reference that is commonly used in the field, though we acknowledge that the other metrics can be used as well. To evaluate the reliability of these descriptors within our statistical framework, Supplementary Table 5 presents model fit metrics (R^2^, ICC, and VIF) for SD, SNR, and RMSD, supporting the robustness and validity of the reported results.

We apply our methods to 64-channel dry EEG data. Although we do not expect changes in our main conclusions for other channel counts, further research is required to test this assumption. To the best of our knowledge, there are no barriers in applying the Fingerprint + ARCI and SPHARA methods to EEG data recorded with higher numbers of EEG channels (Fiedler et al., 2022; Ramon et al., 2023). It has already been demonstrated that the Fingerprint method does not depend on the number of electrodes used, their specific layout, or their type (Tamburro et al., 2018). Therefore, we expect direct applicability of our proposed combination of methods in high-density EEG. The application of our proposed combination of methods to very low numbers of channels such as 19 channels in a 10–20 layout would be particularly relevant for an effective de-noising and artifact reduction of infant EEG recordings, which are typically affected by large amplitude fluctuations. However, for a low number of channels, Fingerprint and ARCI may not be fully suitable, and alternatives such as FORCe (Daly et al., 2015) could be explored.

Our methodology is mostly automated and EEG signal preservation oriented. In contrast to other multivariate data decomposition methods, where the underlying datasets are used to generate the components for the data decomposition, in SPHARA the BFs are determined only by the topological information on the EEG sensor setup. Using the SPHARA BFs, spatial filters can be implemented and applied very quickly, allowing for online applications where the temporal phase properties of the data are not affected by this type of filter, which is particularly suitable for filtering spatially uncorrelated sensor noise. A limitation of using the improved SPHARA (AP0 + SPHARA) method for spatially filtering EEG signals relates to the current need for manual labeling of the artifactual high amplitude jumps in the signal. Although the AP0 step successfully reduces spatial smearing of high amplitude jumps, its reliance on manual labeling limits scalability. Future work should focus on automating AP0 detection using amplitude heuristics, statistical models, or supervised learning based on annotated data. The SWT-kurtosis-based algorithm (Shahbakhti et al., 2021) or similar could be adapted and employed for correcting the high amplitude jumps. Alternatively, other spatial cleaning techniques might be used, such as the common spatial pattern (CSP) approach (Wang et al., 2012; Dong et al., 2023), sensor noise suppression SNS (de Cheveigné and Simon, 2008), local spatial analysis (LSA) (Bufacchi et al., 2021), or oversampled temporal projection (OTP) (Larson and Taulu, 2018).

Given that the EEG signal quality and reliability for dry EEG recordings have been demonstrated to be comparable to that of conventional gel-based recordings (di Fronso et al., 2019), we expect that our combination of Fingerprint + ARCI and SPHARA artifact and noise reduction methods would have a good performance also with traditional gel-based EEG recordings, although this should be addressed in future work. Given the similarities in physiological origins and signal characteristics between EEG and modalities such as magnetoencephalography (MEG) and infant EEG, the proposed Fingerprint + ARCI + AP0 + SPHARA pipeline may hold potential for broader applicability, though this remains to be systematically assessed in future work.

While the sample size is limited, this proof-of-concept study aimed to demonstrate the feasibility of the proposed de-noising pipeline under controlled conditions, laying the groundwork for future validation on larger and diverse datasets.

It is finally worth noting that our proposed combination of methods requires only the EEG signals as input, with no need for additional physiological or technical channels. Additional information, such as from accelerometers, might eventually support the automated labeling of the EEG signal jumps for a more rapid application of the AP0 + SPHARA method.

## 6 Conclusion

We introduced an approach for the removal of physiological and non-physiological artifacts and noise from EEG recordings. Our approach is based on a combination of the Fingerprint + ARCI and improved SPHARA methods and was tested on real EEG signals for the first time. The combination of Fingerprint + ARCI and improved SPHARA methods yielded the best performance when compared to the application of these methods individually. We expect that our proposed approach can be successfully applied for the removal of physiological and non-physiological artifacts and noise not only from multichannel adult EEG but also from infant EEG recordings and multichannel MEG data, resulting in signal quality improvements.

## Data Availability

The raw data supporting the conclusions of this article will be made available by the authors, without undue reservation.
