# Supplementary material for: Combination of spatial and temporal de-noising and artifact reduction techniques in multi-channel dry EEG
Source: Front Neurosci. 2025 Jun 27;19:1576954. doi: 10.3389/fnins.2025.1576954 (PMC12247144; doi:10.3389/fnins.2025.1576954)
Supplement: Supplementary file 1 [file Data_Sheet_1.docx]

Supplementary Material

**Supplementary Table 1.** Total duration of artifactual jump periods (AP0) and total duration of analyzed EEG signals for each volunteer, both summed across all EEG channels and expressed in hours. The relative duration of AP0 periods set to zero Volts is expressed as a percentage of the total analyzed EEG signal duration.

| **Volunteer ID** | **1** | **2** | **3** | **4** | **5** | **6** | **7** | **8** | **9** | **10** | **11** | **Mean** | **SD** |
| --- | --- | --- | --- | --- | --- | --- | --- | --- | --- | --- | --- | --- | --- |
| **Duration of AP0 periods [h]** | 0.17 | 0.15 | 0.53 | 0.20 | 0.22 | 0.30 | 0.32 | 0.15 | 0.95 | 0.72 | 0.71 | 0.4 | 0.3 |
| **Total EEG duration [h]** | 26.2 | 29.9 | 30 | 30.6 | 30.4 | 32.5 | 30.7 | 30.3 | 30.6 | 28.5 | 29.7 | 30 | 1.5 |
| **Relative duration of AP0 periods [%]** | 0.7 | 0.5 | 1.8 | 0.6 | 0.7 | 0.9 | 1.1 | 0.5 | 3.1 | 2.5 | 2.4 | 1.3 | 0.9 |
| **Count of total AP0 periods** | 189 | 236 | 247 | 373 | 178 | 179 | 308 | 167 | 661 | 302 | 504 | 303 | 149 |

Supplementary Tables 2-4 present the detailed results of the generalized linear mixed-effects (GLME) models for signal standard deviation (SD), signal to noise ratio (SNR), and root mean square deviation (RMSD), respectively. The tables show the estimated mean values of the analyzed measures for each method: preprocessed signal (Ref), Fingerprint + ARCI (FPA), SPHARA, FPA + SPHARA, FPA + AP0 + SPHARA. In addition, they present results for signals grouped by the body movement tasks: left hand (L), right hand (R), tongue (T), and feet (F). The intercept, i.e., the models’ reference against which the other fixed effects are compared are the preprocessed signal (Ref) for the methods, and left hand (L) for the tasks. Furthermore, the tables show p-values and confidence intervals for each estimate, indicating the range of variation. If p < 0.005, the differences in the values are considered significant.

**Supplementary Table 2.** Results of the GLME model for EEG signal standard deviation (SD). The ‘Estimate (SD)’ column shows the average SD for the intercept (preprocessed signal (Ref), task L) and changes in SD for the other methods (FPA, SPHARA, FPA + SPHARA, FPA + AP0 + SPHARA) and tasks (R, T, F) compared to the intercept. P-values indicate significance (p < 0.05). Abbreviations L, R, T, and F represent left hand, right hand, tongue, and feet movements, respectively. CI lower and CI upper denote the 95% confidence interval limits. The GLME model was fitted using a gamma distribution with a log-link function.

| Fixed effects – conditions | Estimate (SD) | p-value | CI lower | CI upper |
| --- | --- | --- | --- | --- |
| Intercept (Method: Ref; Task: L) | 2.207 | < 0.001 | 2.113 | 2.302 |
| Methods: |  |  |  |  |
| FPA | -0.163 | < 0.001 | -0.181 | -0.145 |
| SPHARA | -0.206 | < 0.001 | -0.224 | -0.188 |
| FPA + SPHARA | -0.368 | < 0.001 | -0.386 | -0.350 |
| FPA + AP0 + SPHARA | -0.452 | < 0.001 | -0.470 | -0.434 |
| Tasks: |  |  |  |  |
| R | -0.021 | 0.009 | -0.037 | -0.005 |
| T | 0.183 | < 0.001 | 0.167 | 0.199 |
| F | 0.011 | 0.186 | -0.005 | 0.027 |
| Random effects | Estimate (SD) |  | CI lower | CI upper |
| Channel | 0.146 | - | - | - |
| Volunteer | 0.146 | - | - | - |
| Error | 0.344 | - | - | - |

**Supplementary Table 3.** Results of the GLME model for EEG signal to noise ratio (SNR). The ‘Estimate (SNR)’ column shows the average SNR for the intercept (preprocessed signal (Ref), task L) and changes in SNR for the other methods (FPA, SPHARA, FPA + SPHARA, FPA + AP0 + SPHARA) and tasks (R, T, F) compared to the intercept. P-values indicate significance (p < 0.05). Abbreviations L, R, T, and F represent left hand, right hand, tongue, and feet movements, respectively. CI lower and CI upper denote the 95% confidence interval limits.

| Fixed effects – conditions | Estimate (SNR) | p-value | CI lower | CI upper |
| --- | --- | --- | --- | --- |
| Intercept (Method: FPA; Task: L) | 2.152 | < 0.001 | 1.542 | 2.762 |
| Methods: |  |  |  |  |
| SPHARA | -0.764 | < 0.001 | -0.918 | -0.609 |
| FPA + SPHARA | 1.772 | < 0.001 | 1.617 | 1.926 |
| FPA + AP0 + SPHARA | 3.247 | < 0.001 | 3.092 | 3.401 |
| Tasks: |  |  |  |  |
| R | 0.275 | < 0.001 | 0.121 | 0.430 |
| T | 0.013 | 0.865 | -0.141 | 0.168 |
| F | 0.344 | < 0.001 | 0.190 | 0.498 |
| Random effects | Estimate (SNR) |  | CI lower | CI upper |
| Channel | 1.531 | - | - | - |
| Volunteer | 0.777 | - | - | - |
| Error | 2.954 | - | - | - |

**Supplementary Table 4.** Results of the GLME model for EEG root mean square deviation (RMSD). The ‘Estimate (RMSD)’ column shows the average RMSD for the intercept (method Ref, task L) and changes in RMSD for the other methods (FPA, SPHARA, FPA + SPHARA, FPA + AP0 + SPHARA) and tasks (R, T, F) compared to the intercept. P-values indicate significance (p < 0.05). Abbreviations L, R, T, and F represent left hand, right hand, tongue, and feet movements, respectively. CI lower and CI upper denote the 95% confidence interval limits. The GLME model was fitted using a gamma distribution with a log-link function.

| Fixed effects – conditions | Estimate (RMSD) | p-value | CI lower | CI upper |
| --- | --- | --- | --- | --- |
| Intercept (Method: FPA; Task: L) | 1.391 | < 0.001 | 1.263 | 1.520 |
| Methods: |  |  |  |  |
| SPHARA | 0.109 | < 0.001 | 0.084 | 0.134 |
| FPA + SPHARA | 0.343 | < 0.001 | 0.318 | 0.368 |
| FPA + AP0 + SPHARA | 0.435 | < 0.001 | 0.410 | 0.460 |
| Tasks: |  |  |  |  |
| R | -0.007 | 0.592 | -0.032 | 0.018 |
| T | 0.156 | < 0.001 | 0.131 | 0.181 |
| F | 0.048 | < 0.001 | 0.023 | 0.073 |
| Random effects | Estimate (RMSD) |  | CI lower | CI upper |
| Channel | 0.275 | - | - | - |
| Volunteer | 0.181 | - | - | - |
| Error | 0.477 | - | - | - |

Supplementary Table 5 summarizes key model performance indicators for standard deviation (SD), signal to noise ratio (SNR), and root mean square deviation (RMSD). R² marginal and conditional reflect variance explained by fixed effects and by the full model, respectively. The intraclass correlation coefficient (ICC) quantifies the variance attributable to random effects (channels and subjects), while mean variance inflation factor (VIF) assesses multicollinearity. High R² values indicate robust model fit; ICC values reflect meaningful consistency, and VIF confirms the validity of the model-based approach. Overall, the results support the robustness of the analysis.

Supplementary Table 5. Performance metrics from mixed-effects models fitted to EEG signal parameters (SD, SNR, RMSD), including R² marginal and conditional, ICC, and mean VIF.

| EEG signal parameters | R² marginal | R² conditional | ICC | Mean VIF |
| --- | --- | --- | --- | --- |
| SD | 0.991 | 0.995 | 0.499 | 1.362 |
| SNR | 0.643 | 0.716 | 0.205 | 1.286 |
| RMSD | 0.980 | 0.986 | 0.302 | 1.286 |

Supplementary Figure 1 presents the average standard deviations (SDs) of the EEG signal for each of the four tasks (L, R, T, and F), computed across all volunteers and channels. The results demonstrate a reduction in SD with the application of advanced cleaning methods, with the lowest SD observed after the FPA + AP0 + SPHARA method (green dots). Notably, for the tongue movement task (T), a larger variability is observed across all methods, indicating higher noise levels or signal instability during this specific condition.


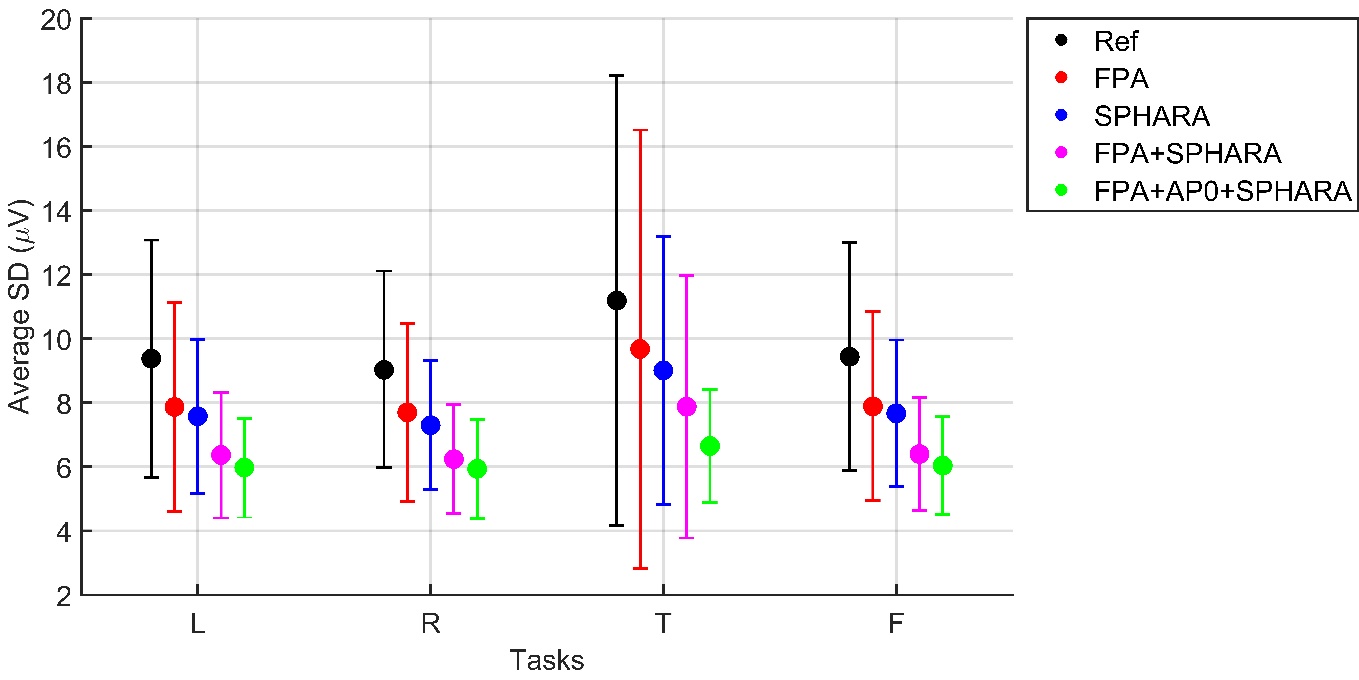


**Supplementary Figure 1.** The average standard deviation (SD) for each task, computed across 11 volunteers and 64 channels. Scatter points represent the average SD values, while error bars indicate the spread of the standard deviation across datasets. The values are calculated after applying each method (Ref, FPA, SP, FPA + SP, FPA + AP0 + SP) and are color-coded, as shown in the figure legend. Abbreviations L, R, T, and F represent left hand, right hand, tongue, and feet movements, respectively.
